# Analysis of the Methylation Level of the *DAT1* Dopamine Transporter Gene in Patients Addicted to Stimulants, Taking into Account an Analysis of Personality Traits

**DOI:** 10.3390/ijms25010532

**Published:** 2023-12-30

**Authors:** Remigiusz Recław, Milena Lachowicz, Krzysztof Chmielowiec, Jolanta Chmielowiec, Aleksandra Strońska-Pluta, Michał Tomasz Kowalski, Bartosz Kudliński, Anna Grzywacz

**Affiliations:** 1 Foundation Strong in the Spirit, 60 Sienkiewicza St., 90-058 Łodz, Poland; health@mocniwduchu.pl; 2 Department of Psychology, Gdansk University of Physical Education and Sport, Kazimierza Górskiego 1 St., 80-336 Gdansk, Poland; milena.lachowicz@awf.gda.pl; 3 Department of Hygiene and Epidemiology, Collegium Medicum, University of Zielona Góra, 28 Zyty St., 65-046 Zielona Gora, Poland; chmiele@vp.pl (K.C.); chmiele1@o2.pl (J.C.); 4 Independent Laboratory of Health Promotion, Pomeranian Medical University in Szczecin, Powstańców Wielkopolskich 72 St., 70-111 Szczecin, Poland; aleksandra.stronska@pum.edu.pl; 5 Clinical Department of Cardiology, Nowa Sól Multidisciplinary Hospital, 67-100 Nowa Sol, Poland; kowaltmd@wp.pl; 6 Department of Emergency Medicine, Anesthesiology and Intensive Care in K. Marcinkowski University Hospital, Collegium Medicum, University of Zielona Góra, 65-046 Zielona Gora, Poland; b.kudlinski@inz.uz.zgora.pl

**Keywords:** dopamine transporter, *DAT1*, methylation, stimulant dependence, NEO-FFI

## Abstract

Drug addiction is a chronic biochemical drug use disorder that affects the human brain and behavior and leads to the uncontrolled use of legal or illicit drugs. It has been shown that three factors are involved in the development of addiction: genetic factors, a diverse environment, and the effect of medication on gene expression. The comprehensive approach and holistic analysis of the problem are due to the multigenic and multifactorial nature of addiction. Dopamine, one of the major neurotransmitters in the brain, is believed to be the “culprit” that leads to a drug abuse-induced “high”. That is why, in our research, we focused mainly on the genes related to dopaminergic reuptake. In the present study, we chose methylation of the *DAT1* dopamine transporter gene based on molecular reasons related to the dopaminergic theory of addiction. This study included two groups: 226 stimulant-dependent and 290 non-stimulant-dependent subjects. The analysis consisted of a case–control comparison of people addicted to psychostimulants compared to a control group of healthy and non-addicted people. There were differences in the levels of statistical significance between the groups. Our research shows lower methylation of islands 1, 9, and 14 in addicted people and greater methylation of islands 32 and 33. The difference in individual CpG methylation islands of the gene under study provides valuable information about the DNA methylation process in patients addicted to psychostimulants. Pearson’s linear correlation analysis in stimulant dependence showed a negative correlation between total methylation island levels and the NEO-FFI Neuroticism scale. In subjects with neuroticism, the methylation level was statistically significantly lower. Pearson’s linear correlation analysis of stimulant-dependent subjects showed a positive correlation between total methylation island levels and the NEO-FFI Openness scale and the NEO-FFI Conscientiousness scale.

## 1. Introduction

Drug addiction is a chronic biochemical drug use disorder that affects the human brain and behavior and leads to the uncontrolled use of legal or illicit drugs. Drug addiction can begin, usually in young people, with non-medical or recreational use of a drug in social settings, which becomes more frequent over time. This involves increasing doses of the drug to achieve a state of euphoria. Drug addiction has been considered a significant social and health problem, posing a threat to public health [1]. In this study, we analyzed patients addicted to psychostimulants, taking into account other factors, including epigenetic and psychological ones, following the holistic approach to the problem of addiction recommended in the literature.

The field of medicine encompasses a category of drugs known as psychostimulants or stimulants. These drugs have been observed to effectively enhance the activity of the central nervous system, thereby conferring numerous benefits to the body [2]. Nevertheless, the widespread and growing use of stimulants has raised public health concerns because of the various physical and mental health risks associated with their use [3]. It is important to note, however, that the category of psychostimulants includes prescription stimulants, including dextroamphetamine and methylphenidate, that are used to treat disorders such as ADHD. These legitimate medications can augment focus, promote sociability, and enhance vigor when taken appropriately under clinical supervision [4]. It has been shown that three factors are involved in the development of addiction: genetic factors, a diverse environment, and the effect of medication on gene expression or mRNA levels [5]. Therefore, an analysis of epigenetic factors, especially those related to methylation, is essential in diseases as complex as addiction [5].

As with other mental disorders, not all people are at the same risk of addiction, and this risk varies significantly from person to person. There are many different factors, characteristics, and variables in the addiction process that determine one’s susceptibility to the development of this disease. In general, the more risk factors a person has, the more likely they are to develop substance use disorder after using drugs occasionally [6]. Recent studies show that transcription factors, including non-coding RNA, histone modifications, and chromatin structure, can alter gene transcription potential. These transcription factors are also important contributors to numerous neuroadaptations that result from chronic drug exposure [7]. Accumulating evidence supports the hypothesis that drugs directly affect all mechanisms of epigenetic regulation and that these adaptations are among the major processes by which drugs induce highly stable brain changes that mediate the phenotype of addiction [8]. Important epigenetic mechanisms are DNA methylation, histone modifications, and small non-coding RNAs [9,10]. Growing evidence has identified the critical mesolimbic dopaminergic circuit as well as essential molecules in this neural pathway that mediate addiction to specific drugs and excessive behaviors [11]. Dopamine, one of the major neurotransmitters in the brain, is believed to be the “culprit” that leads to a drug abuse-induced “high” [12].

That is why, in our research, we focused mainly on genes related to dopaminergic transmission. In the present study, we chose methylation of the *DAT1* dopamine transporter gene based on molecular reasons. The *DAT1* gene, also identified as SLC6A3 [13], is located on chromosome 5p15 and belongs to the Na/Cl-dependent transporter family. This transporter is widely distributed throughout the brain in areas of dopaminergic activity. DAT is located in the plasma membrane of axon terminals and reuptakes dopamine from the synapse [14] and controls the level of dopamine in the extracellular space [15,16,17]. Dysregulated DA activity may result from altered release or reuptake; therefore, the proper regulation of *DAT1* gene expression is crucial for maintaining homeostasis in dopaminergic systems [18].

At the protein level, DAT is present perisynaptically rather than in the synaptic cleft, thus promoting volumetric and somewhat paracrine transmission rather than a standard synaptic transmission that is spatially confined to target postsynaptic dendrites [19]. Interestingly, DAT is present at subcellular levels in intracellular compartments and on the plasma membrane of the dendrites, the cell body, and axon terminals. This suggests that DAT may release DA at the dendritic level near DA cell bodies [20] and that its membrane targeting is regulated by a DA tone. DAT is in the spotlight because of its crucial role in regulating DA transmission, but also because it is a target of psychostimulants such as cocaine and amphetamine [21]. In our study, we linked brain molecular mechanisms related to brain neurotransmission to the epigenetics of addiction.

The dopamine transporter plays an important role in dopamine neurotransmission. It is located on nerve endings and modulates an independent level of dopamine release, returning extracellular dopamine to the presynaptic terminal, thus terminating its function. Impaired dopamine activity may result from altered release or reuptake. For this reason, the regulation of *DAT1* gene expression is important for maintaining homeostasis in the dopaminergic system [18]. There is the possibility, however, that the *DAT1* methylation status might either predispose or reflect exposure to substances in dependent individuals. Therefore, we do not know if it is more of a prognostic or diagnostic marker. However, we do know that, functionally, epigenetic alterations affect the expression of genes, as measured by RNA and protein synthesis; this may affect cellular structure and function and consequently affect the whole body’s metabolism and behavior.

It has been shown that the development of addiction is positively related to genetic factors, suggesting a high heritability of the addiction trait. Drug addiction is not inherited from generation to generation, but, interestingly, personality traits associated with the initiation of drug use are heritable [22]. In addition, epigenetic changes and epigenetic regulators, e.g., chromatin-remodeling enzymes, histone acetyltransferases, and methyltransferases, also play key roles in mediating the long-term effects of drug use [23]. Recent studies have also shown that microRNAs and other non-coding RNAs are essential factors in mediating the rewarding properties of drugs, suggesting that the modulation of post-transcriptional RNA may be a possible pharmacotherapy to reverse drug-induced brain neuroplasticity [24,25].

Recent research has shown that the interaction between genetic and environmental factors and their impact on the emotions and behaviors of children as well as adolescents may depend on the epigenetic mechanisms of DNA methylation [26]. DNA methylation (resulting in gene silencing) is one of the best-studied epigenetic mechanisms. This is mainly accomplished at CpG islands, where cytosine conversion to 5-methylcytosine reduces gene expression. Therefore, in our study, we decided to combine epigenetics with personality traits.

The molecular, cellular, and physiological mechanisms that mediate the transition from sporadic controlled drug use to the loss of control, which, in part, defines addiction, are unknown. However, it is widely believed that changes in gene expression in the central nervous system play a key role [27].

## 2. Results

Differences in methylation levels at individual sites (islands) of the *DAT1* promoter were observed when analyzing the methylation status of unique CpG islands (Table 1). Of the 33 CpG islands, 2 showed significantly higher methylation levels in stimulant dependence (islands 32 and 33) and 3 showed significantly lower levels of methylation in drug addiction (islands 1, 9, and 14). When comparing the odds ratio of increased methylation in the stimulant-dependent group compared to the control group, the islands 1, OR = 1.99; 9, OR = 1.48; 14, OR = 1.87; 32, OR = 0.64; and 33, OR = 0.47 were significant (Table 1).

The analysis of the total methylation of *DAT1* showed a statistically significant difference in the number of total methylated CpG islands in the group with stimulant dependence (41.02%) compared to the controls (42.36%) (Z = −1.172, *p* = 0.2411, Table 1).

While comparing the controls and the group with stimulant dependence, we observed significantly higher scores on the NEO Five-Factor Inventory Neuroticism scale (M 6.58 vs. M 4.61, *p* < 0.00001) and the NEO Five-Factor Inventory Openness scale (M 5.01 vs. M 4.50, *p* = 0.0045). However, significantly lower scores were observed on the NEO Five-Factor Inventory Extraversion scale (M 5.84 vs. M 6.36, *p* = 0.0076), the NEO Five-Factor Inventory Agreeability scale (M 4.28 vs. M 5.59, *p* < 0.00001), and the NEO Five-Factor Inventory Conscientiousness scale (M 5.60 vs. M 6.10, *p* = 0.0173) (Table 2).

**Figure 1:**
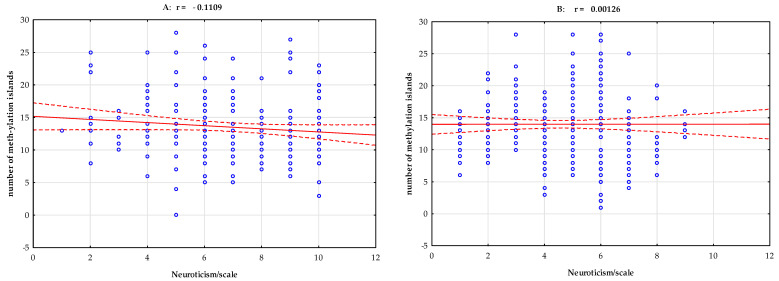
Pearson’s linear correlation between total methylation level, number of methylation islands, and NEO-FFI Neuroticism scale in the group with stimulant dependence (**A**) and the controls (**B**). r—correlation coefficient.

**Figure 2:**
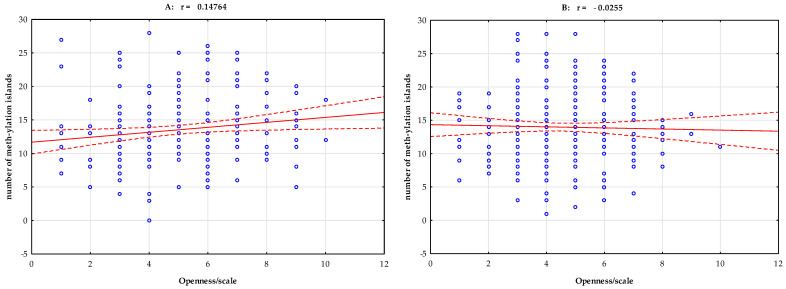
Pearson’s linear correlation between total methylation level, number of methylation islands, and NEO-FFI Openness scale in the group with stimulant dependence (**A**) and the controls (**B**). r—correlation coefficient.

**Figure 3:**
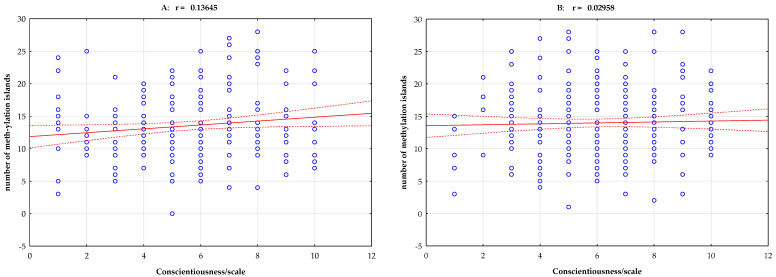
Pearson’s linear correlation between total methylation level, number of methylation islands, and NEO-FFI Conscientiousness scale in the group with stimulant dependence (**A**) and the controls (**B**). r—correlation coefficient.

Pearson’s linear correlation analysis of the group with stimulant dependence showed a negative correlation between total methylation island levels and the NEO-FFI Neuroticism scale (r = −0.154, *p* = 0.020, Figure 1). Pearson’s linear correlation analysis of the group with stimulant dependence showed a positive correlation between total methylation island levels and the NEO-FFI Openness scale (r = 0.148, *p* = 0.026) and the NEO-FFI Conscientiousness scale (r = 0.137, *p* = 0.040, Table 3, Figure 2 and Figure 3).

When analyzing the NEO-FFI Neuroticism scale, we observed a negative correlation with the degree of methylation. On the NEO-FFI Openness scale, a positive correlation was observed in terms of methylation. We also found a positive correlation between the NEO-FFI Conscientiousness scale and methylation. No correlation was observed in the control group.

## 3. Discussion

In the present study, we conducted a methylation analysis in the promoter region of the *DAT1* gene, which included 33 CpG methylation islands. The analysis consisted of a case–control comparison of people addicted to psychostimulants compared to a control group of healthy and non-addicted people. As seen in Table 1, there were differences in the levels of statistical significance between these groups. Interestingly, selected sites in the study group were hypermethylated, and others were hypomethylated compared to the control group. Our research shows lower methylation of islands 1, 9, and 14 in addicted people and greater methylation of islands 32 and 33. As Table 1 shows, total methylation did not differ between the groups. The difference in individual CpG methylation islands of the tested gene provides valuable information about the DNA methylation process in patients addicted to psychostimulants.

Although most studies have focused on histone modifications, DNA methylation is also a critical component of the epigenetic response to psychostimulant-related behaviors. A growing body of research provides evidence for the role of DNA methylation in cocaine-induced neuronal plasticity in the NAc [28,29,30,31]. Several studies have shown that DNMT3A expression levels in the NAc vary with acute and chronic cocaine exposure and during long-term withdrawal, suggesting that psychostimulants are capable of dynamic control of DNA methylation [28,31]. Local knockdown of DNMT3A in the NAc or local infusion of the DNMT inhibitor RG108 enhanced cocaine reward. In contrast, NAc-specific upregulation of DNMT3A attenuated cocaine reward and increased dendritic arborization of NAc neurons [28].

In our study, however, we undertook an even more difficult analysis by adding a factor related to the personality of the studied groups—combining various factors leading to the development of addiction to psychostimulants. We show an interesting result in Table 2—Pearson’s linear correlation analysis of stimulant-dependent subjects showed a negative correlation between total methylation island levels and the NEO-FFI Neuroticism scale (r = −0.154, *p* = 0.020, Figure 1). In subjects with neuroticism, the methylation level was statistically significantly lower. This relationship is also seen in Figure 1. Considering non-biological factors that may influence methylation, we can observe a personality-related factor here. Openness and conscientiousness correlated positively with the methylation of CpG islands in the *DAT1* gene promoter. Pearson’s linear correlation analysis of stimulant-dependent subjects showed a positive correlation between total methylation island levels and the NEO-FFI Openness scale (r = 0.148, *p* = 0.026) and the NEO-FFI Conscientiousness scale (r = 0.137, *p* = 0.040, Table 3, Figure 2 and Figure 3).

This is a difficult aspect for us to discuss because, in research, there is agreement and recommendations for studying psychological and other non-biological traits, but we do not yet know how each of these factors influences the level of DNA methylation in the regions of individual genes. Interestingly, methylation status is widely described in the case of addicts and is sometimes associated with hopes for the selection of therapy. It is increasingly accepted that the overall epigenetic status of a cell can be modulated by various environmental factors, including nutrients, chemicals, and the early life environment [32,33,34]. Early research suggests that various environmental factors also affect brain DNA methylation. For example, repeated SAM (methyl donor) pretreatment significantly potentiated cocaine-induced locomotor sensitization, and the modulatory effect of SAM is due, in part, to reduced methyltransferase activity via the downregulation of DNMT3A [35]. This study supports the hypothesis that environmental factors influencing the epigenetic status of NAc cells may alter how a psychostimulant-induced addiction develops. In addition, these results may at least partially explain why some people are more susceptible to drug addiction.

Here, we can consider whether earlier methylation causes a greater tendency to addiction or whether specific personality traits correlate with epigenetics through genetics. What is interesting, however, is that epigenetic changes are reversible and modifiable.

Because epigenetic mechanisms are dynamic and reversible, chemical agents that alter histone modification or DNA methylation may be potential candidates for therapeutic interventions. In addition, identifying specific epigenetic patterns associated with specific disease phenotypes may provide useful biomarkers for early disease diagnosis and preventative interventions. Future studies should elucidate whether drug effects on epigenetic endpoints in peripheral tissues (e.g., blood) may serve as valuable biomarkers for clinical features of addiction.

The increasing number of reports on drug abuse not only demonstrates the paramount role of epigenetic modifications in regulating behavioral responses to drug exposure but also helps to understand the complex mechanisms underlying drug addiction. However, it is worth noting that only a small number of epigenetic studies of addiction have been conducted in humans. Further research needs to evaluate the possible role of the epigenetic mechanism in addicts [36].

## 4. Materials and Methods

### 4.1. Participants

This study included two groups: 226 stimulant-dependent and 290 non-stimulant-dependent subjects. Table 4 shows the mean age distribution for each group. The study group consisted of men recruited from residential addiction treatment centers. The all-male gender selection was due to the homogeneous gender subgroups of the addiction study (hormonal changes in women, different type and course of addiction, biological and psychological factors influencing the development of addiction). Addiction to illicit substances was reported in the study group.

Our study aimed to analyze a group of patients undergoing treatment in closed addiction treatment centers. A specialist psychiatrist examined the study and control groups, and an interview related to the history of addiction was analyzed. In the current study, patients addicted to psychostimulants constituted a homogeneous subgroup. In the interview with this subgroup, the first addictive substance was amphetamine.

All participants were of European descent to reduce the likelihood of genetic admixture and overcome potential population stratification issues.

The study met the criteria of the Declaration of Helsinki and received a positive opinion from the Bioethics Committee of the Pomeranian Medical University. All participants were informed of the study rules and procedures. In addition, they were informed of their right to withdraw from the study at any time.

None of the participants in the study received any financial incentives for their involvement in the project. The study was entirely anonymized to ensure the protection of personal data. The control group was selected based on age and gender. Throughout the study, all measures were taken to ensure the comfort and concentration of the participants.

A venous blood sample of 9 mL per EDTA tube was collected from the subjects and the genetic material was isolated in the form of DNA.

### 4.2. Assessment of the Methylation Status of the Dopamine Gene Transporter (DAT1) Promoter

According to the manufacturer’s instructions, DNA isolation from peripheral blood was performed using an isolation kit (A&A Biotechnology, Gdynia, Poland). Bisulfite modifications were carried out on 250 ng of DNA using the EZ DNA Methylation Kit from Zymo Research in Orange, CA, USA, following the manufacturer’s instructions. A Mastercycler epgradient S (Eppendorf, Hamburg, Germany) was used for the methylation-specific PCR assay.

Oligonucleotide primers were obtained from Genomed.pl (Warsaw, Poland) and designed using a methprimer (http://www.urogene.org/cgi-bin/methprimer/methprimer.cgi, accessed 29 April 2022). PCRs with primers that targeted the gene fragment were used to evaluate the status of the *DAT1* promoter (ENSG00000142319), i.e., DATF: 5′-GGTTTTTGTTTTTTTTTTGTTGAG-3′; DATR: 5′-AAATCCCCTAAACCTAATCCC-3′. The PCR conditions for amplifying the 447 bp fragment spanning the 33 CpG islands in the *DAT1* gene promoter are shown in Table 5.

The concentration of magnesium chloride ions was set at 2.5 mM. After amplification, PCR products were sequenced as previously described [37]. Briefly, the samples were verified by means of sequencing using the BigDye v3.1 kit (Applied Biosystems, Darmstadt, Germany). The samples were separated by ethanol extraction using ABI Prism 3130XL (Applied Biosystems, Darmstadt, Germany) in a 36 cm POP7 polymeric capillary using a reverse primer.

Sequencing chromatograms were analyzed using 4peaks software (v. 1.8., Mek & Tosj, Amsterdam, The Netherlands, https://nucleobytes.com/4peaks/index.html, accessed 29 April 2023). A G/A + G ratio of at least 20% of the total signal was considered positive for cytosine methylation. The formula for calculating the percentage of methylation in each subject was (G/(G + A) × 100).

### 4.3. Statistical Analysis

To analyze and compare the total methylation levels (%) of 33 CpG *DAT1* islands in the two groups of subjects, the Mann–Whitney U test was used. The personality traits of stimulant-dependent subjects, as measured by the NEO Five-Factor Inventory, were compared with the control group using the same test.

To analyze differences in the methylation percentage at individual CpG islands in the two groups of subjects, chi-squared tests were used, with *p* < 0.05 considered statistically significant.

The relationship between the total methylation levels (%) of 33 CpGs and the personality traits measured by the NEO Five-Factor Inventory was shown separately in both study groups using Pearson’s linear correlation. All statistical analyses were performed using STATISTICA 13 (TIBCO Software, Inc., Palo Alto, CA, USA) and PQStat software (v. 1.8.2., Poznań, Poland).

## 5. Conclusions

Our analysis of the methylation status of individual CpG islands has opened up a new line of research on the biological aspects of psychostimulant addiction. The methylation analysis presented in our study makes it possible to combine the biology of addiction with elements of the analysis of aspects of personality testing. This is an important issue that, for the time being, remains at the level of basic research, but which offers the hope of being used in the future for the individualized treatment of patients, taking into account criteria related to psychology, biology, genetics, and epigenetics.

## Figures and Tables

**Table 1:** Methylation status of 33 CpG *DAT1* islands in the group with stimulant dependence and the control group.

CpG Site	Study Groups Methylation Level n (%)		Test χ^2^	*p* Value	OR	CL −95; +95
	Stimulant Dependence	Controls				
	*n* = 226	*n* = 290				
1	98	175	14.702	0.0001	1.99 *	1.40; 2.83
	43.36%	60.34%				
2	160	225	3.091	0.0787	1.43	0.96; 2.13
	70.80%	77.59%				
3	208	252	3.467	0.0626	0.57	0.31; 1.04
	92.04%	86.90%				
4	51	75	0.747	0.3873	1.20	0.80; 1.80
	22.57%	25.86%				
5	65	97	1.295	0.2550	1.24	0.85; 1.82
	28.76%	33.45%				
6	25	36	0.222	0.6370	1.14	0.66; 1.96
	11.06%	12.41%				
7	28	45	1.023	0.3117	1.30	0.78; 2.16
	12.39%	15.52%				
8	10	12	0.023	0.8794	0.93	0.40; 2.21
	4.42%	4.15%				
9	73	120	4.471	0.0344	1.48 *	1.03; 2.13
	32.30%	41.38%				
10	80	106	0.054	0.8154	1.04	0.73; 1.50
	35.56%	36.55%				
11	7	13	0.654	0.4185	1.47	0.58; 3.74
	3.10%	4.48%				
12	62	86	0.306	0.5799	1.11	0.76; 1.64
	27.43%	29.66%				
13	13	15	0.083	0.7730	0.89	0.42; 1.91
	5.75%	5.17%				
14	181	256	6.566	0.0104	1.87 *	1.15; 3.04
	80.09%	88.28%				
15	182	237	0.118	0.7307	1.08	0.69; 1.69
	80.53%	81.72%				
16	138	185	0.40	0.5246	1.12	0.78; 1.61
	61.06%	63.79%				
17	69	84	0.149	0.6993	0.92	0.63; 1.36
	30.53%	28.97%				
18	12	24	1.722	0.1895	1.61	0.79; 3.29
	5.31%	8.28%				
19	214	276	0.062	0.8038	1.10	0.50; 2.44
	94.69%	95.17%				
20	98	125	0.003	0.9529	0.99	0.70; 1.41
	43.36%	43.10%				
21	158	191	0.951	0.3293	0.83	0.57; 1.21
	69.91%	65.86%				
22	206	265	0.008	0.9272	1.03	0.56; 1.90
	91.15%	91.38%				
23	41	55	0.057	0.8114	1.06	0.67; 1.65
	18.14%	18.97%				
24	153	205	0.535	0.4646	1.15	0.78; 1.68
	67.70%	70.69%				
25	63	93	1.059	0.3035	1.22	0.83; 1.79
	27.88%	32.07%				
26	95	116	0.219	0.6408	0.92	0.65; 1.31
	42.04%	40.00%				
27	41	51	0.027	0.8701	0.96	0.61; 1.52
	18.14%	17.59%				
28	150	198	0.210	0.6470	1.09	0.75; 1.58
	66.37%	68.28%				
29	52	64	0.064	0.7997	0.95	0.63; 1.44
	23.01%	22.07%				
30	27	29	0.498	0.4805	0.81	0.47; 1.43
	11.95%	10.00%				
31	14	16	0.106	0.7442	0.88	0.42; 1.85
	6.19%	5.52%				
32	164	182	5.531	0.0186	0.64 *	0.44; 0.93
	72.57%	62.76%				
33	187	201	12.287	0.0005	0.47 *	0.31; 0.72
	82.74%	69.31%				
			Z	*p*-Value		
Total Methylation Level (%) *	41.02 ± 15.22	42.36 ± 15.70	−1.172	0.2411		
Number of Methylation Islands *	13.54 ± 5.02	13.98 ± 5.18	−1.172	0.2411		

χ^2^ (*p*)—chi-square test (significance level); * differences which are statistically significant (*p* < 0.05); n—number of subjects.

**Table 2:** The results of the NEO Five-Factor Inventory were obtained in steps for the group with stimulant dependence and the control group.

NEO FFI	Stimulant Dependence	Control	Z
	*n* = 226	*n* = 290	(*p*-Value)
Neuroticism/scale *	6.58 ± 2.17	4.61 ± 1.96	9.632 (<0.00001)
Extraversion/scale *	5.84 ± 2.15	6.36 ± 1.99	−2.668 (0.0076)
Openness/scale *	5.01 ± 2.01	4.50 ± 1.60	2.841 (0.0045)
Agreeability/scale *	4.28 ± 1.91	5.59 ± 2.09	−7.001 (<0.00001)
Conscientiousness/scale *	5.60 ± 2.30	6.10 ± 2.14	−2.380 (0.0173)

*p*-value of statistical significance in Mann–Whitney U-test; *n*—number of subjects; M ± SD—mean ± standard deviation; * differences which are statistically significant (*p* < 0.05).

**Table 3:** Pearson’s linear correlation between total methylation level, number of methylation islands, and NEO-FFI in the group with stimulant dependence and the control group.

	Neuroticism Scale	Extraversion Scale	Openness Scale	Agreeability Scale	Conscientiousness Scale
Number of methylation islands	r = −0.154 *	r = −0.021	r = 0.148 *	r = 0.060	r = 0.137 *
Stimulant dependence	(*p* = 0.020)	(*p* = 0.753)	(*p* = 0.026)	(*p* = 0.371)	(*p* = 0.040)
Number of methylation islands	r = 0.001	r = −0.069	r = −0.025	r = 0.084	r = 0.030
Controls	(*p* = 0.983)	(*p* = 0.238)	(*p* = 0.665)	(*p* = 0.155)	(*p* = 0.616)

r—Pearson’s linear correlation; *p*-value of statistical significance; * differences which are statistically significant (*p* < 0.05).

**Table 4:** Primary statistics of analyzed groups.

	Stimulant Dependence	Controls
*n*	226	290
Age M (SD)	27.61 (5.84)	22.17 (4.61)

**Table 5:** PCR conditions for amplification of a 447 bp fragment encompassing 33 CpG islands in the *DAT1* gene promoter.

Number of Cycles	PCR Step	Temperature	Time
1	Initial Denaturation	94 °C	5:00
	Denaturation	94 °C	0:25
35	Annealing	61 °C	0:25
	Elongation	72 °C	0:25
1	Final elongation	72 °C	5:00

## Data Availability

Data are contained within the article.
